# Application of Zeolites and Zeolitic Imidazolate Frameworks in Dentistry—A Narrative Review

**DOI:** 10.3390/nano13222973

**Published:** 2023-11-18

**Authors:** Laura Jiaxuan Li, Chun-Hung Chu, Ollie Yiru Yu

**Affiliations:** Faculty of Dentistry, The University of Hong Kong, 34 Hospital Road, Hong Kong SAR 999077, China; laurali1@connect.hku.hk (L.J.L.); chchu@hku.hk (C.-H.C.)

**Keywords:** zeolite, silver zeolite, zinc zeolite, calcium zeolite, zeolitic imidazolate frameworks, antimicrobial, dentistry

## Abstract

Zeolites and zeolitic imidazolate frameworks (ZIFs) are crystalline aluminosilicates with porous structure, which are closely linked with nanomaterials. They are characterized by enhanced ion exchange capacity, physical–chemical stability, thermal stability and biocompatibility, making them a promising material for dental applications. This review aimed to provide an overview of the application of zeolites and ZIFs in dentistry. The common zeolite compounds for dental application include silver zeolite, zinc zeolite, calcium zeolite and strontium zeolite. The common ZIFs for dental application include ZIF-8 and ZIF-67. Zeolites and ZIFs have been employed in various areas of dentistry, such as restorative dentistry, endodontics, prosthodontics, implantology, periodontics, orthodontics and oral surgery. In restorative dentistry, zeolites and ZIFs are used as antimicrobial additives in dental adhesives and restorative materials. In endodontics, zeolites are used in root-end fillings, root canal irritants, root canal sealers and bone matrix scaffolds for peri-apical diseases. In prosthodontics, zeolites can be incorporated into denture bases, tissue conditioners, soft denture liners and dental prostheses. In implantology, zeolites and ZIFs are applied in dental implants, bone graft materials, bone adhesive hydrogels, drug delivery systems and electrospinning. In periodontics, zeolites can be applied as antibacterial agents for deep periodontal pockets, while ZIFs can be embedded in guided tissue regeneration membranes and guided bone regeneration membranes. In orthodontics, zeolites can be applied in orthodontic appliances. Additionally, for oral surgery, zeolites can be used in oral cancer diagnostic marker membranes, maxillofacial prosthesis silicone elastomer and tooth extraction medicines, while ZIFs can be incorporated to osteogenic glue or used as a carrier for antitumour drugs. In summary, zeolites have a broad application in dentistry and are receiving more attention from clinicians and researchers.

## 1. Introduction

Zeolites are microporous aluminosilicate crystalline materials that can be naturally mined and synthesized. They possess pores and cavities that exchange water, ions and polar molecules with their surroundings [1]. These pores give zeolites ion exchange properties and absorption capacity, allowing them to combine with metal ions to exert antibacterial activities. Zeolites have high chemical stability, thermal stability and biocompatibility [2]. Because of these promising properties, they have been used in a wide range of industrial, agricultural, food and pharmaceutical applications.

Zeolites’ main elements include oxygen, silicon and aluminium. Their structure consists of a three-dimensional framework of silicate [SiO_4_]^4−^ and sluminate. [AlO_4_]^5−^ tetrahedra, connected by shared oxygen atoms [3]. Zeolites’ properties are related to their elemental composition and structure. Pure silica zeolites without aluminium contain silicon in all tetrahedra and have a neutral and stable framework [4]. In contrast, silica zeolite with aluminium components has tetrahedral frameworks that are unbalanced in charge. Zeolites’ polarity decreases with increasing silicon content. Therefore, high-silica zeolites tend to be more thermally and chemically stable and have more hydrophobic surfaces [5], tending to favour low-charge-density (large and monovalent) cations for ion exchange. Low-silica zeolites, on the other hand, are more likely to exchange with high-charge-density (small and multivalent) cations due to their high polarity. Zeolites can be classified based on their silica/aluminium ratio as low-silica zeolites (silica/aluminium ratio below 2), medium-silica zeolites (silica/aluminium ratio between 2~5) and high-silica zeolites (silica/aluminium ratio greater than 5) [6].

**Figure 1:**
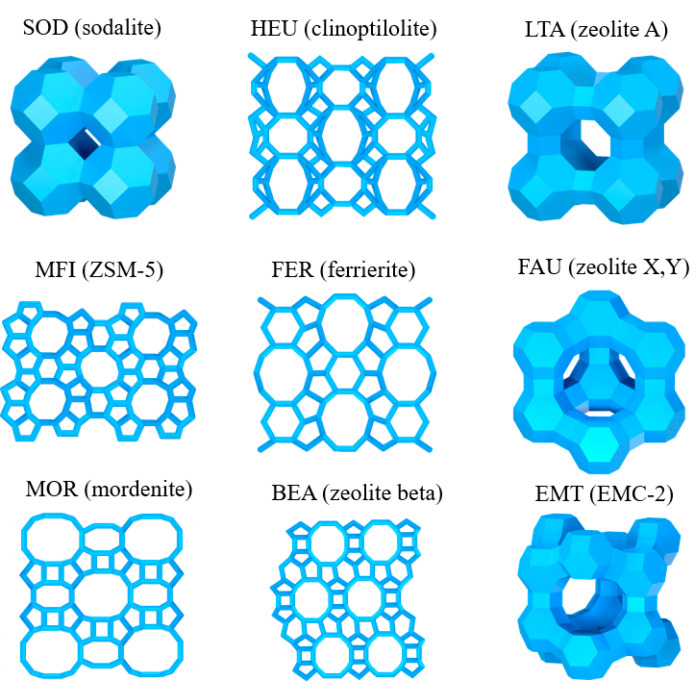
The structure of zeolites [8]. This schematic diagram was made by the authors of the review.

Zeolites present a three-dimensional molecular sieve skeleton structure. The [SiO_4_]^4−^ and [AlO_4_]^5−^ tetrahedral framework is the primary building unit, and these primary units can be arranged to form secondary building unit polycyclic structures, where the zeolite ring usually consists of 4, 5, 6, 8, 10 or 12 tetrahedra [7]. The greater the number of tetrahedra in a single ring, the larger the zeolite’s pore size. The secondary building units are arranged in various geometries to form a composite building unit’s molecular sieve cage structure. The type of skeleton structure of a molecular sieve determines the zeolites’ porosity, pore size and surface area. Structurally, zeolites are classified based on the size of the smallest pores present in the structure as small-pore zeolites (minimum pore size between 3~5 Å, SBU consisting of 8–9 tetrahedra), medium-pore zeolites (minimum pore size between 5~6 Å, SBU consisting of 10 tetrahedra), large-pore zeolites (minimum pore size between 6~7.5 Å, SBU consisting of 12 tetrahedra) and very-large-pore-size zeolites (minimum pore size > 7.5 Å, SBU consisting of >12 tetrahedra) [6]. Zeolites with larger pores and hence higher porosity have a greater ion exchange capacity. Small-pore zeolites include sodalite (SOD), clinoptilolite (HEU) and zeolite A (LTA); medium-pore zeolites include ZSM-5 (MFI) and ferrierite (FER); and large pore zeolites include zeolite X, Y (FAU), mordenite (MOR), zeolite beta (BEA) and EMC-2 (EMT) (Figure 1).

Zeolitic imidazolate frameworks (ZIFs) are a type of Metal–Organic Framework (MOF), which have nano-/microporous structures consisting of metal ions and organic units [9]. Compared with other MOFs, ZIFs have higher thermal, chemical, and water stability [10], and are suitable for biomaterial applications as a carrier for drugs or metal ions. ZIFs are composed of metal ions (e.g., Fe, Co, Cu, Zn) and organic units that are connected by imidazolates. The structure is topologically isomorphic to zeolites [11]. Their metal–imidazolium–metal angle is similar to the 145° Si-O-Si angle in zeolites [12].

ZIFs can be classified according to their topological zeolite-like structure into POZ (ZIF-95), RHO (ZIF-11, ZIF-12, ZIF-71), LTA (ZIF-20, ZIF-21, ZIF-76), SOD (ZIF-8, ZIF-67, ZIF-90, ZIF-91), GME (ZIF-68, ZIF-69, ZIF-70, ZIF-78, ZIF-80, ZIF-82), MER (ZIF-60), DFT (ZIF-3), ANA (ZIF-14), and GIS (ZIF-6, ZIF-74, ZIF-75) [13]. Different synthetic routes and experimental conditions allow the formation of ZIFs with different structures. These types of ZIFs combine the properties of MOFs and zeolites [14]. Taking the SOD-structured ZIF as an example, SOD-ZIF possesses the same structure as SOD-zeolite; however, the pore sizes of 11.6 Å [15] of SOD-ZIF are much larger than the 2.8 Å of SOD-zeolite [16]. The large pore size implies that ZIFs possess stronger ion exchange capacity and adsorption capacity, which make it a very promising material [17].

Due to the distinctive architecture of zeolites and ZIFs, these materials are closely linked with nanomaterials. The presence of micro- or nano-pores within their structure enables the encapsulation of nanoparticles, thereby providing a diverse array of functionalities. Zeolite and ZIFs’ physical and chemical properties and structure make them promising materials for dental applications. The research on the application of zeolites and ZIFs in dentistry has gradually increased in recent years. Previous reviews on application of zeolites in dentistry focused on the available materials and did not cover metal derivatives of zeolites. There is also a lack of discussion on the use of ZIFs in dentistry. Therefore, the aim of this review is to provide an overview of the application of zeolites and ZIFs in dentistry.

## 2. Literature Search

**Figure 2:**
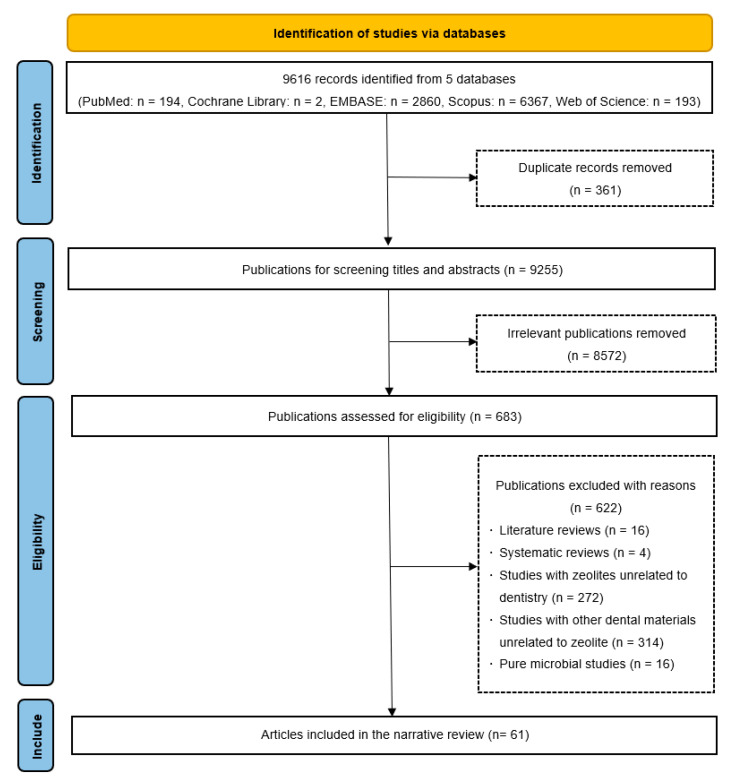
Flow diagram of the literature search and study selection.

We performed a systematic search in five common databases, namely PubMed, Cochrane Library, EMBASE, Scopus and Web of Science. In the search, the keywords used were ((zeolite) OR (ZIF)) AND ((dentistry) OR (dental material)). This review includes all publications on the application of zeolites and ZIFs in dentistry. The included studies were limited to articles published in English on or before 1 October 2023. We removed duplicate articles. We excluded studies on zeolites in fields other than dentistry, microbial studies irrelevant to dentistry or oral health, abstracts, conference papers, literature reviews and systematic reviews. We ultimately included 61 articles in this narrative review. Figure 2 presents the study selection process.

## 3. Zeolites for Dental Application

Zeolite compounds for dental applications are mostly combined with metals or metal derivatives. The zeolite compounds in dental materials are silver zeolite, zinc zeolite, calcium zeolite and strontium zeolite.

### 3.1. Silver Zeolite

Silver zeolite has an aluminosilicate framework containing silver. Its antibacterial properties are mainly derived from the release of silver [18]. The silver can be in various forms, namely silver ions, charged silver clusters and metallic silver nano ions [19]. The distribution pattern of silver depends on the zeolite’s structure and the silicon-to-aluminium ratio. Silver zeolite’s chemical stability contrasts with its cation exchange capacity. The stronger the cation exchange capacity, the easier it is for silver ions to dissociate from the zeolite framework for aggregation or exchange with other cations [19]. The amount of silver ion released is related to the zeolite’s specific surface area and pH value [20]. Silver zeolite has good biosafety and has been used in food preservation as well as disinfection of medical devices and materials [21].

### 3.2. Zinc Zeolite

Zinc zeolites include zinc-cationic zeolites and zinc-oxide zeolites. In general, zinc zeolites have strong stability because zinc has a stabilizing effect on the metal–zeolite system [22]. Zinc zeolites have antibacterial and anti-inflammatory properties and osteogenic activity. Zinc zeolites’ antibacterial properties come from the release of zinc ions [23]. The generation of reactive oxygen species (ROS), including hydrogen peroxide, hydroxyl radicals and superoxide ions [24], also contributes to zinc zeolites’ antibacterial properties.

### 3.3. Calcium Zeolite

Calcium zeolite has a stable particle size and molecular sieve shape and steadily releases calcium ions. In the oral environment, calcium zeolite can deliver calcium ions to the tooth surface, rebuild the hydroxyapatite structure of dentin and enamel, and fill in the gaps where hard tissue demineralization occurs due to bacteria-generated acid, thus showing remineralization potential [25]. In addition, the combination of calcium ions and zeolite can enhance the physical adsorption of zeolite [26]. Apart from the calcium ion zeolite, the zeolite–hydroxyapatite material also releases calcium ions and has a remineralization potential [27].

### 3.4. Strontium Zeolite

Strontium zeolite can release Sr^2+^ ions sustainably. It can promote dentin remineralization. Strontium ions (Sr^2+^) can replace Ca^2+^ in the apatite structure in the dental hard tissue and bone tissue to promote the proliferation and differentiation of human dental pulp stem cells [28].

## 4. ZIFs for Dental Application

The most commonly used ZIFs in dentistry are ZIF with SOD structures, including ZIF-8 and ZIF-67.

### 4.1. ZIF-8

ZIF-8 consists of zinc ions (Zn^2+^) and 2-methylimidazole (2-MIM). It has the advantages of a large surface area with a porous structure, low density, high thermal stability, strong resistance to hydrolysis [29], high biocompatibility [30,31], and stable release of zinc ions [32]. Zinc ions released from ZIF-8 promotes bone regeneration by up-regulating the expression of osteogenesis-related genes and osteogenic proteins [33], activates multiple osteogenesis pathways and activates growth factors [34]. ZIF-8 has pH-responsive properties. ZIF-8 is stable in water and alkaline solutions but breaks down rapidly in acidic environments [35].

### 4.2. ZIF-67

ZIF-67 consists of cobalt ions (Co^2+^) and 2-methylimidazole (2-MIM), which also has the advantages of large surface area, controlled pore size, good biocompatibility, and biodegradability [36]. ZIF-67 is also pH-responsive. ZIF-67 is stable under neutral conditions, whereas under acidic conditions, ZIF-67 can rapidly decompose and release Co^2+^ [37,38].

## 5. Dental Applications of Zeolites and ZIFs

Zeolites have been employed in various areas of dentistry, such as restorative dentistry, endodontics, prosthodontics, implantology, periodontics, orthodontics and oral surgery. In restorative dentistry, zeolites are used as antimicrobial additives in dental adhesives, temporary filling materials and restorative materials. In endodontics, they are used in root-end fillings, root canal irritants, root canal sealers and bone matrix scaffolds. In prosthodontics, zeolites can be incorporated into denture bases, tissue conditioners, soft denture liners and dental prostheses. In implantology, zeolites are applied in dental implants, bone graft materials, bone adhesive hydrogels, drug delivery systems and electrospinning. In periodontics, they can be applied as antibacterial agents for deep periodontal pockets, guided tissue regeneration membranes and guided bone regeneration membranes. Zeolites are also used in orthodontic appliances in orthodontics and in oral cancer diagnostic marker membranes, maxillofacial prosthesis silicone elastomer, osteogenic glue and tooth extraction medicines for oral surgery (Table 1).

### 5.1. Restorative Dentistry

Silver zeolite and zinc zeolite have been used to enhance the antimicrobial properties of adhesives and restorative materials. Calcium zeolite can be relied upon for its antimicrobial and remineralising properties, which protect the tooth structure by reducing the removal of deep carious tissue and minimising the risk of pulpal exposure. In addition, restorative materials modified with zeolites and ZIFs are mechanically stronger than conventional resin-based materials and are more conducive to bonding system stability.

#### 5.1.1. Zeolite/ZIF-Modified Adhesives

Zeolites have been used to modify dental adhesives. Zeolites containing zinc and silver can be added with dental adhesive to improve their antibacterial properties, biocompatibility and wettability, thereby improving the long-term bonding strength between resin and dentin [39].

ZIFs have also been used to improve the dental adhesives’ viscoelasticity [40,41], adhesion strength [41] and thermal stability [42]. The zinc ions in ZIF-8 can inhibit the hydrolytic degradation of collagen fibres in dentine, which enhances the strength of the resin–dentin interface and prolongs the service life of adhesives and the bonded dental fillings [43].

#### 5.1.2. Zeolite-Loaded Restorative Materials

Zeolites have been loaded into restorative materials to improve their antimicrobial and physical properties. The zeolite-loaded resin-based restorative material presented a lower amount of bacterial attachment than unmodified resin [44]. In addition, the wettability of zeolite-loaded restorative materials was lower, indicating decreased solubility [44]. Calcium zeolites can improve the restorative materials’ remineralising properties by providing calcium ions sustainably to the dental hard tissue [27,45]. Silver–zinc zeolite was added to the temporary filling material to inhibit the growth of Streptococcus pyogenes, Streptococcus pneumoniae, Streptococcus salivarius and Streptococcus haematogenic through the stable release of silver and zinc ions [46].

### 5.2. Endodontics

In endodontics, silver zeolite with antimicrobial properties can be added in root-end fillings, root canal irrigants, and root canal sealers. Zeolite compounds and ZIF-modified materials with antimicrobial and anti-inflammatory properties show potential in endodontic application. Further studies are needed to investigate the irritating effects of zeolite compounds and ZIFs on the dental pulp.

#### 5.2.1. Zeolite-Incorporated Materials for Root-End Fillings

Silver zeolite-incorporated mineral trioxide aggregate, a root-end filling material, has shown antibacterial activity against Enterococcus faecalis [47]. However, the addition of zeolites decreases the material’s compressive strength and push-out bond strength [48,49].

#### 5.2.2. Zeolite-Incorporated Materials for Root Canal Irrigants

Silver zeolite-incorporated root canal irrigants can inhibit the formation of biofilms of Enterococcus faecalis, Staphylococcus aureus and Candida albicans [50].

#### 5.2.3. Zeolite-Incorporated Materials for Root Canal Sealers

Silver zeolite-incorporated root canal sealers increased adhesion to dentin [51] and can provide better filling capacity for complex anatomical root canal structures [52]. It can also effectively inhibit the adherence of Enterococcus faecalis [53], Streptococcus miller and Staphylococcus aureus [54,55].

### 5.3. Prosthodontics

In prosthodontics, silver zeolite and zinc zeolite can be added to dental prostheses, tissue conditioners, denture bases, and soft denture liners to exert antimicrobial effects, enhance the bond strength of restorations, and increase the surface hardness and smoothness of denture bases. However, the effect of zeolites on the physical properties of the materials has not been accurately determined, and further research is thus needed.

#### 5.3.1. Zeolite-Infiltrated All-Ceramic Dental Prostheses

The zeolite-infiltrated all-ceramic prosthesis enhanced its aesthetic properties and adhesive properties [56]. This prosthesis showed better aesthetic performance because the alumina in the zeolite effectively intercepted the incoming light, weakening the light transmission [56]. Zeolite-infiltrated all-ceramic laminated veneers enhanced the bonding strength to the inner core and prevented the veneer from chipping off [57]. Because zeolite-infiltrated all-ceramic veneers present similar thermal expansion properties with the porcelain dental core, it reduces stress concentrations due to mismatches in the coefficients of thermal expansion, thereby increasing the bond strength of the two. In addition, zeolites do not affect the infiltrated prostheses’ inherent mechanical properties.

#### 5.3.2. Zeolite-Incorporated Tissue Conditioners

Silver-zeolite-incorporated tissue conditioners present an antibacterial effect against Candida albicans, Staphylococcus aureus and Pseudomonas aeruginosa [58]. The addition of silver zeolite does not affect the inherent dynamics of viscoelasticity tissue conditioners [59].

#### 5.3.3. Zeolite-Loaded Denture Bases

The acrylic resin denture base loaded with silver–zinc zeolite has a stronger antibacterial effect [60,61], a higher surface hardness and a smoother surface than the conventional denture base [62]. In addition, zeolites increase the denture base’s opacity, which may have an aesthetic impact [60]. However, the addition of zeolites reduces the denture base’s deformation resistance and impact strength [63].

#### 5.3.4. Zeolite-Incorporated Soft Denture Liners

The addition of silver–zinc zeolites to the soft denture liner enhances its antimicrobial effect and physical strength [64]. Soft denture liners loaded with Ag-Zn zeolite nanoparticles have a long-term antifungal effect than those with fluconazole [65].

### 5.4. Implantology

In implantology, strontium zeolite, ZIF-8 and ZIF-67 can provide antimicrobial and osteogenic properties in dental implant-related materials. Current materials used for bone regeneration and reconstruction have limitations that prevent them from combining mechanical strength, biocompatibility and osteogenic activity at the same time. ZIFs present corrosion resistance, good antimicrobial properties, high biocompatibility, and the ability to induce bone mineralisation and regeneration, making them suitable when used as coatings on implants and scaffolds. The application of zeolites and ZIFs in implantology brings more options for clinical treatments.

#### 5.4.1. Zeolite/ZIF-Coated Implants

Titanium dental implants coated with strontium zeolite show enhanced biocompatibility, corrosion resistance, osteogenesis and osseointegration [66]. Zinc zeolite-coated implants showed enhanced bone cell activity and promoted osteogenesis and bone integration [67], reducing the risk of implant loosening [68,69]. In addition, zeolites combined with silver ions, zinc ions or other metal ions have strong antibacterial properties and great potential when applied in dental and bone implants [70]. ZIF-8- and ZIF-67-coated dental and orthopaedic titanium implants have strong antimicrobial properties, corrosion resistance and biocompatibility [71,72].

#### 5.4.2. ZIF-Coated Bone Graft Materials

ZIF-8 can modify biphasic calcium phosphate ceramics (BCP), a bone graft material, by coating the surface. The ZIF-8-coated BCP altered the ceramics’ surface chemistry and can effectively promote cell attachment, proliferation, osteogenic differentiation and bone regeneration [73].

#### 5.4.3. ZIF-Modified Bone Adhesives

The bone adhesive modified with ZIF-8 nanoparticles has strong antibacterial properties, wet adhesion, crosslinking density and mechanical strength. It performs better than normal bone adhesives in stabilizing the environment of bone grafts, promoting osteogenic differentiation and preventing the deformation and collapse of bone grafts under external forces [74].

#### 5.4.4. ZIF-Loaded Drug Delivery System

The drug delivery system loaded with ZIF-8 nanoparticles more effectively promoted periosteum vascularization and vascular coupling than drugs not loaded with zeolites in the treatment of extensive bone defects. The half-life of the drug such as desferriamine or dimethyloxallyl glycine in the system was prolonged, preventing its rapid clearance in plasma [75,76].

#### 5.4.5. ZIF-Modified PCL Electrospinning

The electrospinning of polycaprolactone (PCL) modified with ZIF-8 nanoparticles has the potential to promote bone regeneration in implant surgery by inducing neovascularization. Moreover, it has the advantages of high porosity, stable physical properties, slow release of zinc ions and a controlled degradation rate [77].

#### 5.4.6. ZIF-Modified Post-Implantation Drugs

The manganese-doped ZIF-8 can inhibit over-reactive inflammation and prevent wound infections after implant surgery [78]. It releases manganese ions and zinc ions simultaneously, presenting both bactericidal and anti-inflammatory functions.

### 5.5. Periodontics

In periodontics, silver zeolite and ZIF-8-loaded deep periodontal pocket drugs can reduce the bacterial load and alleviate inflammation. They are promising in both the basic and surgical treatment stages of periodontal disease. ZIF-embedded guided tissue/bone regeneration membranes can promote periodontal tissue and alveolar bone regeneration.

#### 5.5.1. Zeolite/ZIF-Loaded Drugs for Deep Periodontal Pockets

The silver-zeolite-loaded drug for deep periodontal pockets inhibited common Gram-negative bacteria, including *Pseudomonas gingivalis, Pseudomonas intermedia* and *Pseudomonas actinomyces*, and Gram-positive bacteria pathogenic bacteria under anaerobic conditions. Therefore, it can be used as an antibacterial drug for patients with periodontitis [79].

ZIF-8-loaded deep periodontal pocket drugs have antibacterial and anti-inflammatory effects [80,81] and osteogenic properties, which can promote alveolar bone regeneration in patients with periodontal bone loss [82].

#### 5.5.2. ZIF-Embedded Guided Tissue/Bone Regeneration Membranes

A functional guided tissue regeneration (GTR) membrane coated with ZIF-8 nanoparticles has a porous structure and randomly oriented nanofibers, which can effectively prevent cell migration across the membrane barrier [83]. Zinc ions’ antibacterial action can prevent bacterial infection after GTR.

A guided bone regeneration (GBR) membrane coated with ZIF-8 nanoparticles has good antibacterial properties, an asymmetric porous structure and suitable porosity and pore size, which are conducive to the growth of bone tissue [84]. In addition, the ZIF-8 nanoparticles’ modified GBR membrane is conducive to the primary attachment, growth and proliferation of dental pulp stem cells [85]. However, the modified film’s mechanical properties were slightly reduced.

### 5.6. Orthodontics

In orthodontics, zinc-oxide zeolite is used to modify orthodontic brackets to provide an antibacterial effect and prevent dental plaque attachment around the brackets.

#### 5.6.1. Zeolite-Modified Orthodontic Brackets

Zeolite can be used to modify the orthodontic bracket in orthodontic treatment, mainly as an antibacterial agent. The orthodontic bracket modified with zinc-oxide zeolite has a strong antibacterial effect against *Klebsiella pneumoniae* and *Escherichia coli* [86]. However, the bracket’s bending strength decreases with the increase in zinc-oxide zeolite concentration.

#### 5.6.2. Zeolite-Based aPDT Photosensitizer

Antimicrobial photodynamic therapy (aPDT) is a method for orthodontic bracket cleaning. This method uses light to activate photosensitizers that produce ROS to kill bacteria. Zinc-oxide zeolite-based aPDT photosensitizers have a strong bactericidal effect on the cariogenic microbial biofilm formed on the orthodontic bracket. Additionally, they promote remineralisation on the demineralised enamel around the bracket [87].

### 5.7. Oral Surgery

In oral surgery, zeolite and ZIFs are mainly used for post-extraction treatment, cranial and maxillofacial bone restoration, and oral oncology. Zeolites can be used to promote resorption and bone healing in post-extraction sockets. Zeolites and ZIF-8 can be added to bone glue and maxillofacial silicone elastomers to increase their mechanical strength and promote bone regeneration. ZIF-8 can be used to transport antitumour drugs. In addition, currently ZIFs have the potential to be designed as stimulus-responsive drug delivery systems to stimuli including light, heat, and magnetism [88]. They can also be used as immune checkpoint inhibitors, immune adjuvants or carriers of cancer vaccines for the immunotherapy of tumours [89].

#### 5.7.1. Zeolite-Modified Bone Matrix Scaffold

A bone matrix scaffold modified by nano-zeolites has improved osteogenic properties, compressive strength and surface hardness compared to an unmodified scaffold [90,91]. A bone matrix scaffold modified by nano-zeolites promotes bone formation by promoting the adhesion of calcium and phosphate ions and apatite crystallization [92]. Zeolite nanoparticles have strong interaction with plasma proteins [93]. Silicon in zeolites plays an important role in the formation of hard tissue in the early stage of bone calcification [94]. Some zeolites can also increase intracellular ALP activity, thereby enhancing the proliferation and osteogenic reaction of human dental pulp stem cells [90].

#### 5.7.2. Zeolite-Loaded Oral Cancer Detection Membrane

A detection membrane loaded with zeolites can be used for the diagnosis of oral cancer [95]. The detection membrane analyses the volatile organic compound (VOC) spectrum of the patient’s saliva and diagnoses the condition based on the potential markers of oral squamous cell carcinoma displayed on the VOC spectrum. However, the authors of this study did not explain the role of zeolites in the detection membrane.

#### 5.7.3. Zeolites Act as a Drug after Tooth Extraction

Zeolites (clinoptilolite) can be used as a drug after tooth extraction to promote wound healing and new bone formation [96]. Due to zeolites’ ion exchange and absorption properties, they can detoxicate the socket by irreversibly absorbing bacteria, histamine and other inflammatory proteins and exudates in the socket. They can also attract blood clots in the socket and promote the formation of granulation tissue, thus easing wound inflammation and promoting healing. Zeolites also provide calcium and silica, which are essential minerals for bone formation.

#### 5.7.4. Zeolite-Modified Maxillofacial Silicone Elastomers

The incorporation of silver–zinc zeolite into maxillofacial silicone elastomers enhances their mechanical properties, allowing the material to better withstand mechanical loading [97].

#### 5.7.5. ZIF-Incorporated Osteogenic Glue

Osteogenic glue is used to repair fractured bone or dislocated teeth. The ZIF-8-incorporated osteogenic glue and its osteogenic effect was conducive to an increase in bone thickness at the fracture site and the growth of new bone tissue [98].

#### 5.7.6. ZIF-Coated Antitumour Drugs

Antitumour drugs coated with ZIF-8 nanoparticles have a higher tumour inhibition rate than uncoated drugs. ZIF-8 can be degraded in the acidic tumour microenvironment, thereby providing better drug delivery [99].

## 6. Conclusions

Zeolites and ZIFs are crystalline aluminosilicates with porous structure, which are closely linked with nanomaterials. Their structure enhances ion exchange capacity, physical–chemical stability and biocompatibility, making them promising materials for dental applications. The common zeolite compounds for dental applications include silver zeolite, zinc zeolite, calcium zeolite and strontium zeolite, while the common ZIFs for dentistry include ZIF-8 and ZIF-67. Zeolites and ZIFs have been applied in multiple areas of dentistry and show great potential in their application in clinical dental practice.

## Figures and Tables

**Table 1:** Types, properties, functions and applications of zeolites/ZIFs in dentistry.

Dental Application of Zeolites	Type of Zeolite/ZIF	Properties of Zeolites in Materials	Functions of Zeolites in Materials
**Restorative Dentistry**			
Zeolite/ZIF-modified adhesives	Silver–zinc zeolite ZIF-8	Offer antimicrobial properties Improve bonding strength and shear strength	Prevent secondary caries Prolong lifespan of restoration
Zeolite-loaded restorative materials	Silver–zinc zeolite Calcium zeolite	Offer antimicrobial properties Improve bonding strength Improve corrosion resistance	Prevent secondary caries Promote the remineralization of demineralized tooth tissue
**Endodontics**			
Zeolite-incorporated root filling	Silver zeolite	Offer antimicrobial properties	Prevent root canal reinfection
Zeolite-incorporated irrigants	Silver zeolite	Offer antimicrobial properties	Prevent root canal reinfection
Zeolite-incorporated sealers	Silver zeolite	Offer antimicrobial properties	Prevent root canal reinfection
**Prosthodontics**			
Zeolite-infiltrated all-ceramic dental prostheses	Zeolite (sodalite)	Enhance material aesthetics Improve bonding strength	Improve material optical properties Prevent the veneer chipping off
Zeolite-incorporated tissue conditioners	Silver zeolite	Offer antimicrobial properties	Prevent candida stomatitis
Zeolite-loaded denture bases	Silver–zinc zeolite	Offer antimicrobial properties Improve surface hardness and smoothness	Prevent denture stomatitis
Zeolite-incorporated denture liners	Silver–zinc zeolite	Offer antimicrobial properties	Prevent denture stomatitis
**Implantology**			
Zeolite/ZIF-coated implant	Strontium zeolite ZIF-8 ZIF-67	Offer antimicrobial properties Enhance osteogenic activity	Prevent infection after implant surgery Promote bone differentiation and regeneration Prevent implant loosening
ZIF-coated bone graft materials	ZIF-8	Enhance osteogenic activity	Promote bone differentiation and regeneration
ZIF-modified bone adhesive	ZIF-8	Offer antimicrobial properties Improve wet adhesion and mechanical strength Enhance osteogenic activity	Prevent infection after implant surgery Prevent the deformation of bone graft Promote bone differentiation and regeneration
ZIF-loaded drug delivery system	ZIF-8	Enhance osteogenic activity Prolong half-life period of drug	Promote periosteal vascularization Promote bone differentiation and regeneration
ZIF-modified PCL electrospinning	ZIF-8	Enhance osteogenic activity	Promote angiogenesis Promote bone differentiation and regeneration
ZIF-modified post-implantation drug	ZIF-8	Offer antimicrobial properties	Prevent wound infections after implant surgery
**Periodontics**			
Zeolite/ZIF-loaded deep periodontal pocket drugs	Silver zeolite ZIF-8	Offer antimicrobial properties Enhance osteogenic activity	Prevent and treat periodontitis Promote bone differentiation and regeneration
ZIF-embedded guided tissue regeneration membranes	ZIF-8	Offer antimicrobial properties Enhanced barrier action Enhance osteogenic activity	Prevent infection after GTR Promote periodontal tissue regeneration
ZIF-embedded guided bone regeneration membranes	ZIF-8	Offer antimicrobial properties Enhance osteogenic activity	Prevent infection after GBR Promote bone differentiation and regeneration
**Orthodontics**			
Zeolite-modified orthodontic bracket	Zinc-oxide zeolite	Offer antimicrobial properties	Reduce plaque attachment around brackets
Zeolite-based PDT photosensitizer	Zinc-oxide zeolite	Offer antimicrobial properties	Reduce plaque attachment around brackets
**Oral surgery**			
Zeolite-modified bone matrix scaffold	Zeolite	Enhance osteogenic activity	Promote bone differentiation and regeneration
Zeolite-loaded oral cancer detection membrane	Zeolite (ZSM-5)	Not mentioned	Improve detection accuracy
Zeolite acted as the drug after tooth extraction	Zeolite (clinoptilolite)	Offer absorption property Provide essential minerals	Promote wound healing Promote bone formation
Zeolite-modified maxillofacial silicone elastomer	Silver–zinc zeolite	Enhance mechanical properties	Prevent material breakage or degradation
ZIF-incorporated osteogenic glue	ZIF-8	Enhance mechanical strength and hard-tissue adhesion Enhance osteogenic activity	Promote bone formation
ZIF-coated antitumour drugs	ZIF-8	Degrade in acidic environment	Improve drug transportation and volatilization efficiency

## Data Availability

Not applicable.
